# Conversion and Tack-Curing of Light-Cured Veneer Luting Agents

**DOI:** 10.3390/jfb16090307

**Published:** 2025-08-26

**Authors:** Aikaterini Petropoulou, Maria Dimitriadi, Spiros Zinelis, Ioannis Papathanasiou, George Eliades

**Affiliations:** 1 Department of Fixed Prosthodontics, School of Dentistry, National and Kapodistrian University of Athens, 2 Thivon Str., 11527 Athens, Greece; johnpapatha@dent.uoa.gr; 2 Department of Biomaterials, School of Dentistry, National and Kapodistrian University of Athens, 2 Thivon Str., 11527 Athens, Greece; mardimit@dent.uoa.gr (M.D.); szinelis@dent.uoa.gr (S.Z.); geliad@dent.uoa.gr (G.E.)

**Keywords:** veneer luting agents, degree of conversion, tack-curing, FTIR spectroscopy, hardness

## Abstract

Light attenuation and excess handling of light-cured luting agents create problems in bonding veneer restorations. The aim of the present study was to assess the curing capacity of light-cured veneer luting agents (VLA) [Choice 2 (CH2), G-Cem Veneer (GCV), Panavia LC Veneer (PNV), PermaCem LC Veneer (PMS), and Variolink Esthetic LC (VEV)] under lithium disilicate veneers, in the presence or absence of touch-cure primers (Adhese Universal Bond DC for VEV, G Premio Bond + DCA Activator for GCV, and V5 Tooth Primer V5 for PNV) and to evaluate material setting under two tack-curing irradiation modes (contact, distant). The methods used were ATR–FTIR spectroscopy and Vickers hardness (VHN) tests (*n* = 5/product and test). According to the results, all materials cured under the ceramic demonstrated significantly lower DC% from the controls, with a ranking of VEV, CH2 > GCV, PMS, PNV. The primers improved DC% by 4–13% only in the veneer groups. Tack-curing in contact mode demonstrated conversion and hardness values ranging from 37% to 78% and 31% to 57% of the controls respectively, corresponding to a vitreous state. For the distant mode, very low conversion values were found (0–7% of the controls), with untraceable indentations. It can be concluded that low translucency veneers reduce conversion of VLAs, which can be improved by using touch-cure activators. Tack-curing, as instructed, creates vitrified materials, resulting in difficult removal of set excess, which implies the need for better standardization of the procedure.

## 1. Introduction

Ceramic veneers are considered among the most popular treatments in aesthetic dentistry. This type of restoration requires minimal preparation, preserves tooth structure, demonstrates strong bonding with dental hard tissues, has excellent soft-tissue compatibility, and offers exceptional esthetics [1]. Nevertheless, several technical (chipping, fracture, and debonding) and biological (secondary caries) complications have been described for this delicate treatment modality [2].

The bonding technique is a key factor for the longevity of ceramic veneers [3]. Unlike full contour restorations that rely on retentive design, veneers bond with teeth through micromechanical interlocking and chemical adhesion. For etchable, glass-ceramic materials (porcelain and lithium disilicate), hydrofluoric (HF) acid-etching and silanization are considered the best adhesive treatment, whereas for the stronger polycrystalline zirconia, sandblasting (alumina or alumina/silica) and use of primers (phosphate and silane/phosphate monomers) are advocated [4,5]. The veneers are bonded to acid-etched enamel with resin-based luting agents, which play a critical role in determining the overall strength, marginal adaptation, and longevity of the restoration [6].

Translucent restorations, augmented with the optical characteristics of the natural dentition, are very sensitive to the intrinsic color stability of the luting agents. For such cases, light-cured materials stand out as the preferred choice, due to their superior optical properties, the extended working time that allows precise adjustment of the restoration, and the enhanced degree of conversion [7,8,9,10,11].

Apart from the initial color change upon irradiation due to photobleaching of camphorquinone in light-cured systems [12], the color of these materials is more stable than the versions incorporating amine-peroxide self-curing catalysts [11]. Although the Vanadium-activated hydroperoxide–thiourea systems available in modern dual-cured resin luting agents have improved color stability [13,14], light-cured materials are still dominating this application. An additional advantage of light-cured veneer luting agents is the absence of adhesive monomers, contrary to many dual-cured adhesive and self-adhesive luting agents. The presence of many chromophore groups in adhesive monomers provides an additional source of color instability [15].

Due to the external principle of photoactivation, the curing capacity of the light-cured luting agents is affected by the wavelength, intensity, and beam homogenization of the light emitted by the curing unit; the exposure time; the transmission parameter and thickness of the veneer materials; and the composition of the luting agents [16]. To address these challenges, manufacturers have improved resin formulations by introducing reactive dimethacrylate monomers with high crosslinking capacity [17], adjusting filler composition, size, and shape [18], and refining photoinitiator systems. These developments enhanced curing efficiency, improved the mechanical, chemical, and biological performance of the restorations [19,20], and reduced polymerization shrinkage, which is important for the marginal integrity of the restorations [21,22].

Since the bonding of ceramic veneers to phosphoric acid-etched enamel by modern light-cured luting agents is mediated by adhesive primers or universal adhesives, the possibility of interferences cannot be excluded. Moreover, excess removal is an important and challenging clinical step in the overall bonding procedure. Tack-curing, by momentary irradiation of the excess material to reach the gel state and then removal by a sharp probe, is the most common method for easy and effective marginal cleaning [23]. Several studies are available for the effect of tack-curing on the overall setting behavior of light-cured luting agents. It has been postulated that tack-curing (2–5 s) reduces superficial hardness in some materials, interfering thus with the setting reaction [24], while it provides fewer marginal defects in comparison with removal of the unset luting agent by a brush prior to setting [25]. Nevertheless, the results of other studies reached contrary conclusions, but for dual-cured materials [26].

The aim of the present study was (a) to assess the curing capacity of light-cured veneer luting agents with and without the presence of a glass-ceramic veneer, (b) to evaluate whether the use of adhesives affects the curing capacity of the veneer luting agents, and (c) to investigate the conversion and hardness of the excess material after tack-curing. To address these issues, three experiments were performed. The null hypotheses were (a) there are no differences in the curing capacity of the materials with or without the ceramic veneers, (b) the use of adhesives does not affect conversion of the luting agents, and (c) there are no differences in the properties of the luting agents after tack-curing.

## 2. Materials and Methods

The light-cured veneer luting materials tested are presented in Table 1.

### 2.1. Degree of Conversion Under the Ceramic Veneers

**Figure 1:**
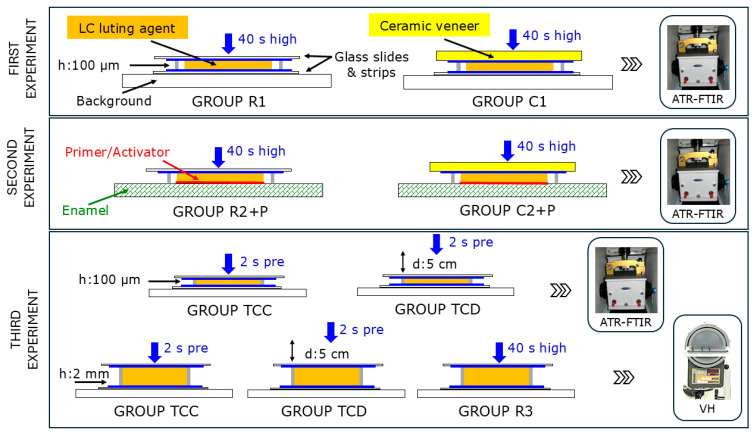
The testing procedures employed in the three experiments performed (High: high intensity curing and Pre: tack-curing).

Metallic spacers of 100 μm thickness were used to prepare disc-shaped veneer composite specimens (Ø = 5 mm, *n* = 5/material), enclosed between microscopic glass slides covered with Mylar strips. The specimens were placed on a white background surface with 80% reflectance and directly irradiated for 40 s with an LED curing unit (Bluephase G4, Ivoclar Vivadent) emitting 1200 mW/cm^2^ light intensity (“high” irradiation mode), as measured with a curing radiometer (Bluephase 2, Ivoclar Vivadent). This group served as a control (Group R1). Another group (Group C1, cured under ceramic) was prepared by placing over the top Mylar strips of 0.7 mm thick lithium disilicate glass–ceramic specimens (Ø: 6 mm, Emax-Press, shade LT-A2, *n* = 5/material) and light-cured as previously described (Figure 1, 1st experiment). The total transmittance of the ceramic discs was ≈45% as measured with an integrated sphere (RSA-PE-20, PerkinElmer, Norwalk, CT, USA) attached to a UV–Vis spectrometer (Lambda 35, PerkinElmer). The specimens were stored for 10 min at 37 °C (dark/dry conditions).

The degree of conversion (DC%) of the luting agents was measured by attenuated total reflection FTIR spectroscopy (ATR–FTIR) at top specimen surfaces. An ATR accessory was used (Golden Gate, Specac, Oprington, Kent, UK) with a single-reflection diamond element (2 × 2 mm) and ZnSe lenses, attached to an FTIR spectrometer (Spectrum GX, PerkinElmer, Buckinghamshire, Bacon, UK). Spectra were acquired under the following conditions: 4000–650 cm^−1^ wavenumber range, 4 cm^−1^ resolution, 20 scans co-addition, and ≈2 μm depth of analysis at 1000 cm^−1^. Spectra of uncured composite pastes served as reference. For the DC% measurements, the aliphatic C=C stretching vibrations (1635 cm^−1^) were chosen as analytical bands (AN), whereas the aromatic C=C stretching vibrations (1605 cm^−1^ for CH2, GCV, PMS, and VEV) or the N–H bending vibrations at 1535 cm^−1^ (for PNV) were selected as the reference bands (RF). Quantification was performed according to the equation: DC% = 100 × [1 − (ApAN × AmRF/AmAN × ApRF)], where A is the net peak absorbance height of set (p) and unset (m) materials in analytical (AN) and reference (RF) bands, respectively.

### 2.2. Degree of Conversion in the Presence of Touch-Cure Activators

Specimens (100 μm thick) were prepared from GCV, PNV, and VEV luting agents, as in Group R1, by replacing the bottom glass slide and strip with polished human enamel surfaces (IRB: 675/20-1-2025), which were etched with 37% orthophosphoric acid gel for 15 s, water-rinsed, air-dried, and treated with the corresponding primers/adhesives (G-Premio Bond + DCA Activator for GCV, V5 Tooth Primer for PNV, and Adhese Universal DC for VEV), according to the manufacturers’ instructions, to assess any positive effect of touch-cure adhesives and activators on luting agent conversion (Figure 1, 2nd experiment). The specimens were prepared with (Group C2+P, *n* = 5/material) and without the ceramic veneers as above (Group R2+P, *n* = 5/material) and stored for 10 min at 37 °C (dark/dry conditions). The DC% at the top specimen surfaces was measured by ATR–FTIR as previously described.

### 2.3. Effect of Tack-Curing on Setting Characteristics

(a) Degree of conversion: Specimens (100 μm thick, *n* = 5/material) prepared as per Group R were irradiated with the light-curing unit set in the “Pre” mode (2 s, 950 mW/cm^2^), and the DC% was measured on the directly irradiated surface immediately after irradiation with the light-curing tip in contact with the glass (Group TCC). A second specimen series was prepared and irradiated under the same conditions but with the curing tip placed at 5 cm distance from the top glass (Group TCD), corresponding to a calculated intensity of 380 mW/cm^2^ (Figure 1, 3rd experiment). The DC% measurements were performed as described before (Section 2.1).

(b) Hardness: Metallic rings of 2 mm thickness were used to prepare disc-shaped specimens (Ø = 5 mm, *n* = 5/material and group) enclosed between microscopic glass slides covered with Mylar strips and irradiated, employing (a) the “Pre” curing mode (2 s at 950 mW/cm^2^) with the curing tip in contact with the top glass (Group TCC), (b) the “Pre” curing mode with the curing tip at 5 cm distance from the top glass (Group TCD), and (c) the “high” curing mode (40 s at 1200 mW/cm^2^) with the curing tip in contact with the top glass (Group R3). Immediately after irradiation, the Vickers hardness of the top specimen surfaces was measured with a hardness tester (Diatronic 2RC, Wolpert, Ludwigshafen, Germany) under a 1 kp load, for a 10 s contact period, and at 70× objective. Two measurements were performed on each surface 1 mm from the margins, from which an average value was obtained representing each specimen (Figure 1, 3rd experiment).

### 2.4. Statistical Analysis

The number of specimens used per test were based on the previous literature on DC% and VH measurements. Shapiro–Wilk and Brown–Forsythe tests were used to assess the normal distribution and equal dispersity of the tested populations respectively. A 2-way (independent factors: “material” and “veneer”) and a 3-way analyses of variance (independent factors: “material”, “veneer”, and “primer”)—ANOVA—were employed to assess the effect of ceramic veneer on DC% and the effect of individual primers on DC% in the presence or absence of the veneers. For tack-cured specimens, the DC% data failed normality test for 2-way ANOVA. Therefore, 1-way ANOVA on Ranks was used for comparisons within the TCD group, and 1-way ANOVA was used within the TCC group and among groups for the same material. For hardness measurements, the data failed equal variance test for 2-way or split 1-way ANOVA. Consequently, 1-way ANOVA on Ranks was used for comparisons within TCC and R3 groups, and 1-way ANOVA for comparisons between groups per material. Multiple comparison tests (Holm–Sidak or Tukey) were used to allocate pair differences. All analyses were performed at a 95% confidence level (α = 0.05) using the Sigma Plot v.15 software (Systat Software Inc., San Jose, CA, USA).

## 3. Results

**Figure 2:**
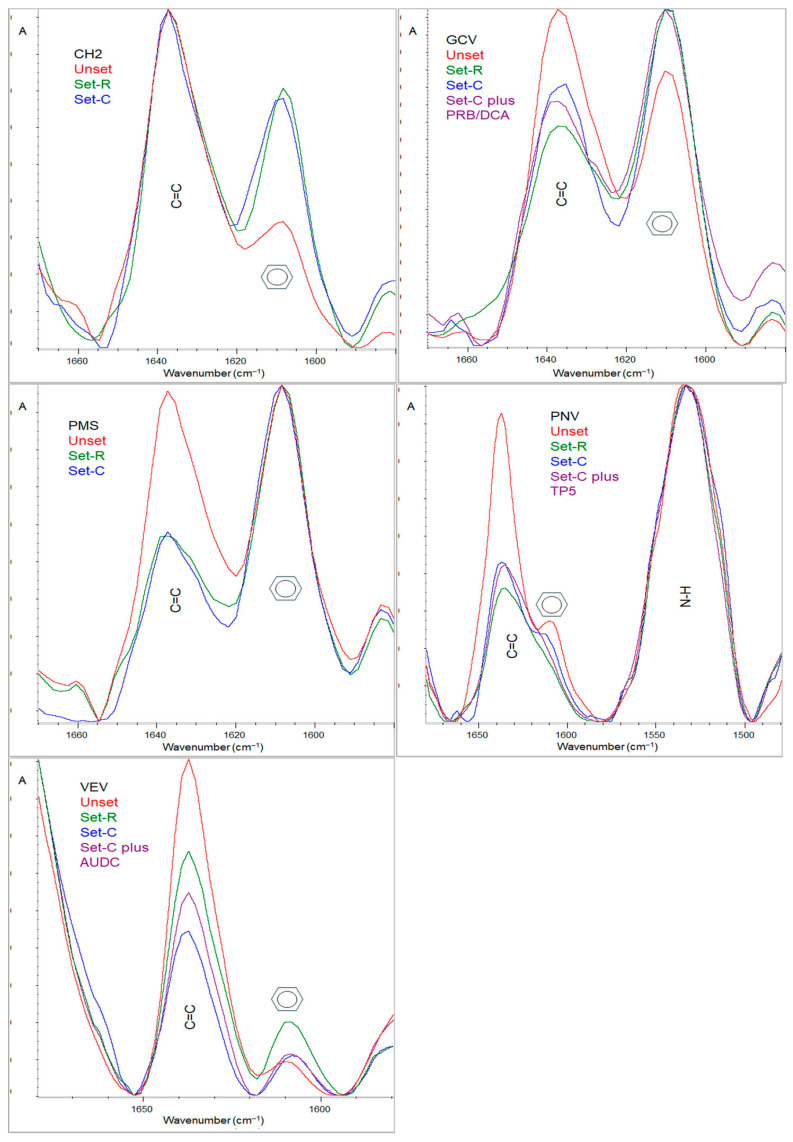
Representative ATR–FTIR spectra of luting agent specimens with the corresponding bands used for calculation of DC% of unset, reference set (R), set under veneers (C), and set under veneers treated with primers/adhesives (C plus the corresponding material).

Representative ATR–FTIR spectra with the corresponding analytical and reference bands used for DC% measurements in the first (Section 2.1) and second (Section 2.2) experiments are illustrated in Figure 2. All materials demonstrated well-defined peaks of aliphatic and aromatic vibrations, except for PNV, where a minor aromatic peak appeared in the unset group, which after irradiation became a weak broad shoulder to the main C=C peak at 1638 cm^−1^. For this reason, the N-H peak at 1535 cm^−1^ was used as the reference band.

### 3.1. Degree of Conversion Under the Ceramic Veneers

The quantitative results of the DC% derived from the first experiment are summarized in Table 2. The results passed normality (Shapiro–Wilk, *p* = 0.287) and equal variance tests (Brown–Forsythe, *p* = 0.593). The 2-way ANOVA failed to properly identify a main effect since a significant interaction was found between the factors “material” and “veneer” (*p* = 0.029). All materials demonstrated significantly lower DC% values when irradiated under the veneers (Group C1) in comparison with direct irradiation (Group R1). Among the materials, insignificant differences were found among GCV-PNV and PMS-PNV (Group C1) and CH2-VEV, GCV-PNV, GCV-PMS, and PMS-PNV (Group R1).

### 3.2. Degree of Conversion in the Presence of Touch-Cure Activators

The results of DC% where the veneers and controls were irradiated on primed enamel, along with the data obtained on glass substrate (as listed in Table 2 for comparison), are presented in Table 3. The data for the 3-way ANOVA (independent factors: “material”, “veneer”, and “primer”) passed normality (*p* = 0.101) and equal variance tests (*p* = 0.460). Significant interactions were found for the factors “material” and “veneer” (*p* = 0.011) and “veneer” and “primer” (*p* < 0.001), but not between “material” and “primer” (*p* = 0.430). For the factor “veneer”, significant differences were found for all materials in favor of the control group (R1). The use of the primers provided significantly higher DC% only in the veneer group (Group C2+P, *p* < 0.001).

### 3.3. Effect of Tack-Curing on Setting Characteristics

(a) Degree of conversion: The DC% data, along with the data obtained on glass substrate without primers (as listed in Table 2 for comparison), are presented in Table 4. Within each material group, the ranking of the significant differences was R1 > TCC > TCD. Within the TCC group, significant differences were found among CH2-GCV, PNV, and PMS-PNV, while within the TCD group, the ranking was CH2, VEV > GCV, and PMS > PNV.

(b) Hardness: The results of VHN (Table 4) confirm the superior performance of the control (Group R3) vs. TCC. Hardness measurements were not applicable in Group TCD, since it was impossible to observe an indentation due to the fluid or elastic state of the materials. Significant differences were found among PMS-PNV, PMS-CH2, and GCV-PNV (Group TCC) and CH2-PNV, CH2-VEV, and PMS-PNV (Group R3).

## 4. Discussion

Light-cured luting agents are mainly used for bonding of transparent or semi-transparent laminates or crowns, where the colors of the agent and the substrate are integrated with the restoration. In such cases the activating light can be transmitted through the restoration at adequate intensity levels to effectively polymerize the underlying material. In the present study, 0.7 mm thick low translucency (45%) lithium disilicate glass ceramics were used as veneer material, representing more challenging light-curing conditions from high translucency veneers, along with five modern light-cured veneer luting agents of different monomer, filler, and catalyst content.

The first experiment compared the degree of conversion of the luting agents after direct irradiation, considered as standard, and after irradiation through the veneers. In the current study, a polywave LED curing unit was used, which, due to the reflector design, provides a homogeneous beam profile, to ensure absence of spatial intensity deviations as observed with many polywave curing units [27]. Although 20 s of irradiation was instructed by most manufacturers, 40 s irradiation was used to succeed acceptable conversion levels, considering the relatively low translucency of the ceramic used. VEV is a BisGMA-free material. However, clear aromatic peaks appeared in the ATR–FTIR spectra, which implies that there are aromatic components in the material with a peak intensity clearly resolved to be used for quantification. The study confirmed previous findings on the reduced conversion obtained under the veneers due to attenuation of the activating light [28]. Nevertheless, the reduction in DC% ranged from 9% to 12% in comparison with the control, which indicates a rather limited effect. Post-curing time is known to enhance DC% from 2% to 18%, dependent on the initial exposure time and irradiance [29,30]. Considering that the exposure time used was two times higher than the instructed, a limited extent of post-curing conversion is anticipated in the current experiment. Differences in DC% between materials are mainly attributed to the monomer reactivity [31], filler [32], and catalyst content [33]. In accordance with a previous study [34], VEV provided the highest DC% under optimal conditions (control, Group R1) since it is free of BisGMA monomers that possess high steric hindrance, has low filler loading avoiding interference with conversion, and contains photoinitiators with high quantum yield (i.e., Ivocerin). Only one product (PMS) was based on traditional BisGMA/TEGDMA monomer composition. In all other products, UDMA has been used as comonomer with BisGMA (apparently at the expense of BisGMA) or with other bisphenol or bisphenol-free adducts to improve conversion and flexibility [35]. Alternative monomers have been also introduced in these materials to improve conversion and curing rate (DDDMA and NPGDMA) and increase strength, stiffness (NPGDMA and THFMA), and crosslinking (NPGDMA and GDMA) [36,37,38,39], thus providing a balance of optimized structure–property relationship. When polymerization was performed under the veneers, the ranking of significant differences in DC% was changed for CH2 and GCV, indicating lower and higher sensitivity to light reduction, respectively.

In clinical practice, the light-cured luting agents are placed on conditioned and primed dental surfaces. Therefore, questions are raised about the development of interfacial interactions between primers/adhesives and luting agents relevant to conversion. This task was addressed in the second experiment, where the primers/adhesives were tested on enamel, a substrate that can efficiently neutralize the acidic components, facilitating adhesive conversion [40]. It has been well-documented that in slow-setting materials (i.e., self-curing or dual-curing without light exposure), acidic primers and adhesives interfere with the setting reaction due to the protonization of the amine component of the conventional benzoyl peroxide–amine catalysis systems [41]. Such incompatibility issues have been resolved by the addition of aryl sulfinate activators to the adhesives, transforming the materials to dual-cured, or by using transient metal activators in dedicated primers or adhesives of universal resin luting agents [42,43]. For light-cured materials, no incompatibility problems have been identified when light-curing proceeds within a reasonable period after the composite has been placed in contact with the adhesive, except for cases of very high acidic monomer concentrations, which deviate from the current applications in the field [43]. It has been also been well-established that the use of sulphinates, borates, or transient metal activators exert a touch-cure effect, increasing the conversion of the tissue-infiltrated adhesive fraction [44], creating a free-radical gradient from the interface towards the material, which may improve conversion in the adjacent thin layers of luting agents and core build-up materials [43,45]. The sulphinate activators of GCV (in the DCA Activator vial) and VEV (on the microbrush tip of Adhese Universal DC) are known to enhance polymerization of camphorquinone–amine systems when used as co-catalysts, without inducing discoloration problems [46]. Conversely, the catalysts of PNV are based on the color-stable amine-free hydroperoxide–thiourea system, accelerated by Vanadium compounds, which are incorporated in the luting agent and the corresponding primer (V5 Tooth Primer) [14]. In the current study, no positive effect was experienced in DC% of directly irradiated groups with the primers (R2+P) because the DC% measured in the control was already high and the improvement potential was diminished. However, a 4–13% increase in DC% was registered when the primers were used for the veneer group (C2+P). The small improvement in DC% may not significantly enhance the mechanical, chemical, or biological performance of the restoration, but reduction of residual C=C bonds, which are active chromophore groups [47], may offer higher color stability.

Tack-curing has been introduced as a fast, efficient, and reliable method for the removal of excess luting material from the restoration margins before the final curing [48]. However, the technique has not been standardized yet, with various versions having been instructed by manufacturers regarding the irradiation time (1–5 s), the profile and distance of the irradiating tip (standard or mini-lens type and placed close or distant to the material), and the curing intensity (standard or reduced). All these may affect the extent of the polymerization of excess material and consequently the cleaning capacity and marginal quality of the restorations [48]. The aim of tack-curing is to tack the veneer in place, avoiding shifting, and provide a solid, rubbery consistency setting to the excess (gel state), which can be then easily removed with a sharp instrument, without creating marginal artifacts to the main restoration (i.e., undercuts, marginal gaps, etc.). In the present study, the LED light-curing unit used provided a special setting for tack-curing (“Pre” irradiation mode: 2 s, 950 mW/cm^2^), which was used for all materials. Since the intensity was considered quite high, two sets of measurements were performed, in contact (TCC) and 5 cm distant (TCD) to the top material surface covered with the glass slide and cellulose strip. The DC% values of the TCC group ranged between 62% and 78% of their controls (Group R1), except for PNV, which reached 37% of the control, implying that it is less sensitive to activating light. These values obtained immediately after irradiation were quite high for providing a rubbery state phase. This was confirmed by hardness measurements, where values ranging between 31% and 57% of the controls were recorded. In all cases, straight sides of Vickers indentations were depicted with absence of recovery to support the development of a rubbery state. Under these conditions, the excess cannot be easily removed, and removal may affect the resinous phase at the veneer margins. When measurements were performed with the light-curing tip at 5 cm distance (380 mW/cm^2^ estimated intensity), no hardness measurements could be obtained since the materials did not set. Probing with a dental explorer revealed minor signs of rubbery state development only in CH2, which showed a DC% around 37% of the control. In all other materials, which exhibited very low DC% values (0–7% of their controls), no resistance to probing could be detected. Although the effect of tack-curing on the properties of dual- and light-cured luting cements has been investigated in several studies [24,26,48,49], the issue of excess removal has received limited attention. An in vitro test of PNV excess removal in tack-curing mode (5 s with an undefined curing unit and intensity) presented fewer marginal defects but greater marginal discoloration in comparison with the brushing technique [25]. In another study on VEV, tack-curing for 1 or 5 s with two light-curing units (1600 and 1470 mW/cm^2^ light intensity) provided a rather complex pattern of differences from the brushing technique [50]. A trend for higher removal capacity after tack-curing was observed with insignificant differences among the excess removal method, marginal resin defects and microleakage. The authors of the study highlighted the difficulty in removing the tack-cured excess, especially after 5 s exposure. For the highly reactive light-cured luting agents, irradiation at high intensity even for the shortest period of 1s makes it rather impossible to produce a semi-gel or rubbery state, even when polymerization is performed in air and material setting is inhibited to a depth of few microns. The gel state in conventional dimethacrylate dental monomers starts at very low conversion (<10%), while vitrification is developing at ≈40% conversion [51]. As observed in the present study, the dedicated tack-curing mode of the modern light-curing unit used exceeded the upper gel-state limit, producing vitreous phases with well-defined Vickers hardness (10–28 VHN) reaching 31–57% of the hardness of fully polymerized controls. These values correspond to soft materials (like PMMA) but with no rubbery consistency, as considered to be the requirement of tack-curing. Under these conditions, excess removal from the margins of not fully-bonded veneers is a challenging procedure to avoid implication of marginal defects in the luting resin. Distant irradiation in the tack-curing mode seems much safer but, for the current curing unit, should be performed at closer distance to allow proper excess gelation. These findings highlight the need for further studies on the standardization of the tack-curing process to facilitate the important clinical step of excess removal from veneer restorations. It should be mentioned that data for tack-curing derived from dual-curing luting agents, especially the self-adhesive ones, should not be used as a guide for the light-cured luting agents since the presence of acidic monomers modifies the setting behavior of these products.

The results of the present study render the first null hypothesis invalid since significant differences were found in the DC% among material groups cured with or without the veneers and within each material group between the two conditions. The second null hypothesis should be partially rejected because significant differences were found in DC% in the veneer group when primers with touch-cure activators were used. Finally, the third null hypothesis should be rejected owing to the significant differences encountered in DC% and VHN among the tack-cured modes and materials tested.

## 5. Conclusions

The materials irradiated at high intensity under the low translucency veneers demonstrated significantly lower DC% from the controls. The ranking in significant differences of the controls was VEV > CH2 > GCV > PNV and PMS and transformed to VEV, CH2 > GCV, PMS, and PNV under the veneers. The use of touch-cure primers with the corresponding materials improved DC% by 4–13% only in the veneer groups. Tack curing in contact mode demonstrated conversion and hardness values ranging from 37% to 78% and 31% to 57% of the controls, respectively, corresponding to a vitreous state. For the distant mode, very low conversion values were found (0–7% of the controls), with untraceable indentations.

## Figures and Tables

**Table 1:** The light-cured veneer luting agents tested.

Materials	Composition *	Manufacturer
Choice 2 (CH2)	UDMA, BisGMA, TEGDMA, THFMA, catalysts, BaAlBSiO_4_-glass, YbF_3_, SiO_2_ (filler: 50–75%wt)	Bisco, Schaumburg, IL, USA
G-Cem Veneer (GCV)	UDMA, NPGDMA, BisEMA, TCDDMDMA, TEGDMA, methylated melamine-formaldehyde polymer, catalysts, pigments (filler: 69%wt)	GC Int., Tokyo, Japan
Panavia Veneer LC (PNV)	UDMA, TEGDMA, hydrophilic aliphatic dimethacrylate, hydrophilic amide monomer, catalysts, pigments YbF_3_, silanated spherical SiO_2_ (filler: 66%wt, 47%vol, size 0.05–8 μm)	Kuraray Noritake, Okuyama, Japan
Permashade LC Veneer (PMS)	BisGMA, TEGDMA, catalysts, pigments (filler: 66.5%wt)	Ultradent Int. S. Jordan, UT, USA
Variolink Esthetic LC (VEV)	UDMA, DDDMA, GDMA, catalysts, pigments, spheroid mixed oxide, YbF_3_ (filler: 38%vol, size 0.04–0.2 μm, mean 0.1 μm)	Ivoclar Vivadent Schaan, Lichtenstein

* According to manufacturers’ information. BisEMA: ethoxylate bisphenol glycidyl dimethacrylate, BisGMA: bisphelol glycidyl dimethacrylate, DDDMA: dodecanediol dimethacrylate, GDMA: glycerol dimethacrylate, NPGDMA: neopentylglycol dimethacrylate, TEGDMA: triethyleneglycol dimethacrylate, TCDDMDMA: tricyclodecane dimethanol dimethacrylate, THFMA: tetrahydrofurfuryl methacrylate, and UDMA: urethane dimethacrylate.

**Table 2:** The results of DC% for the specimens irradiated under the ceramic veneers (C1) and the directly irradiated controls (R1).

	Materials				
Groups	CH2	GCV	PNV	PMS	VEV
C1	64.6 (2.3) a, A	58.5 (2.0) a, B	56.7 (1.5) a, B	57.9 (0.9) a, B	66.7 (1.7) a, A
R1	71.7 (2.4) b, A	65.5 (2.1) b, B	64.1 (2.7) b, B, C	61.7 (1.6) b, C	76.1 (3.7) b, D

Means and standard deviations. Same letters indicate insignificant differences (*p* > 0.05) between groups per material (lowercase) and among materials within the same group (uppercase). All significant differences were at the *p* < 0.001 level.

**Table 3:** The results of DC% for the specimens irradiated under the ceramic veneers (C) and the directly irradiated controls (R) on enamel with (+P) and without primer/adhesive application.

	Materials		
Groups	GCV	PNV	VEV
C2+P	65.9 (2.9) a, A	60.8 (1) a, B	69.5 (2.6) a, C
C1	58.5 (2.0) a, B	56.7 (1.5) a, B	66.7 (1.7) a, A
R2+P	64.7 (2.6) a, A	64.9 (3.1) a, A	75.7 (2.7) c, B
R1	65.5 (2.1) b, B	64.1 (2.7) b, B, C	76.1 (3.7) b, D

Means and standard deviations. Same letters indicate insignificant differences between groups per material (lowercase), and among materials within the same group (uppercase), with *p* < 0.001 for all comparisons, except for GCV vs. PNV within C+P group (*p* = 0.003) and C+P vs. C groups within PNV (*p* = 0.02).

**Table 4:** Results of DC% and VH of tack-cured materials in contact with the top surface (TCC) at 5 cm distance (TCD) and the corresponding controls (R1 for DC% and R3 for VH).

	Materials				
Groups	CH2	GCV	PNV	PMS	VEV
			DC%		
TCC	55.6 (1.2) a, A	40.8 (1.1) a, B	23.8 (2.0) a, C	39.0 (0.9) a, B	52.7 (2.5) a, A
TCD	26.1 (1.2) b, A	3.1 (0.2) b, B	0 b, B	4.1 (0.5) b, C, B	3.8 (0.6) b, B
R1	71.7 (2.4) c, A	65.5 (2.1) c, B	64.1 (2.7) c, B, C	61.7 (1.6) c, C	76.1 (3.7) c, D
			**VHN**		
TCC	16.9 (1.3) a	23.7 (2) a	10 (0.6) a	27.5 (1.5) a	16.9 (1.3) a
TCD	NA	NA	NA	NA	NA
R3	54.3 (2.9) b, A	45.9 (5.8) b, A	20.4 (1.1) b, B	50.6 (4.5) b, A	29.6 (1.1) b, C

Means and standard deviations. Same letters indicate insignificant differences between groups per material (lowercase) and among materials within the same group (uppercase) per property (*p* > 0.05). NA: impossible to measure.

## Data Availability

The original contributions presented in the study are included in the article; further inquiries can be directed to the corresponding author.
