# Correction: Petropoulou et al. Conversion and Tack-Curing of Light-Cured Veneer Luting Agents. *J. Funct. Biomater.* 2025, *16*, 307

**DOI:** 10.3390/jfb17040191

**Published:** 2026-04-15

**Authors:** Aikaterini Petropoulou, Maria Dimitriadi, Spiros Zinelis, Ioannis Papathanasiou, George Eliades

**Affiliations:** 1 Department of Fixed Prosthodontics, School of Dentistry, National and Kapodistrian University of Athens, 2 Thivon Str., 11527 Athens, Greece; johnpapatha@dent.uoa.gr; 2 Department of Biomaterials, School of Dentistry, National and Kapodistrian University of Athens, 2 Thivon Str., 11527 Athens, Greece; mardimit@dent.uoa.gr (M.D.); szinelis@dent.uoa.gr (S.Z.); geliad@dent.uoa.gr (G.E.)

## Error in Table

In the original publication [1], there was a mistake in Table 3 as published. The author stated that there were two rows of data missing in Table 3 (R2+P and C2+P). The corrected Table 3 appears below. The authors state that the scientific conclusions are unaffected. This correction was approved by the Academic Editor. The original publication has also been updated.

## Figures and Tables

**Table 3:** The results of DC% for the specimens irradiated under the ceramic veneers (C) and the directly irradiated controls (R) on enamel with (+P) and without primer/adhesive application.

	Materials		
Groups	GCV	PNV	VEV
C2+P	65.9 (2.9) a, A	60.8 (1) a, B	69.5 (2.6) a, C
C1	58.5 (2.0) a, B	56.7 (1.5) a, B	66.7 (1.7) a, A
R2+P	64.7 (2.6) a, A	64.9 (3.1) a, A	75.7 (2.7) c, B
R1	65.5 (2.1) b, B	64.1 (2.7) b, B, C	76.1 (3.7) b, D

Means and standard deviations. Same letters indicate insignificant differences between groups per material (lowercase), and among materials within the same group (uppercase), with *p* < 0.001 for all comparisons, except for GCV vs. PNV within C+P group (*p* = 0.003) and C+P vs. C groups within PNV (*p* = 0.02).
